# Vacuum-assisted staged omphalocele reduction: A preliminary report

**DOI:** 10.3389/fped.2022.1053568

**Published:** 2022-11-24

**Authors:** Matthias Nissen, Anna Romanova, Elena Weigl, Laura Petrikowski, Mohamad Alrefai, Jochen Hubertus

**Affiliations:** ^1^ Department of Pediatric Surgery, Marien Hospital Witten, Ruhr-University Bochum, Germany; ^2^ Department of Pediatric Surgery, Dr. von Hauner Children's Hospital, Ludwig-Maximilian-University, Munich, Germany

**Keywords:** giant omphalocele, congenital abdominal wall defect, staged omphalocele reduction, vacuum therapy, VAC

## Abstract

**Introduction:**

Omphalocele represents a rare congenital abdominal wall defect. In giant omphalocele, due to the viscero-abdominal disproportion, gradual reintegration of eviscerated organs is often associated with medical challenges. We report our preliminary experience combining staged gravitational reduction with vacuum (VAC) therapy as a novel approach for treatment of giant omphalocele.

**Patients and methods:**

Retrospective chart review of six patients (five females) born between September 2018 and May 2022 who underwent staged reduction of giant omphalocele in conjunction with VAC therapy was conducted. Treatment was performed at two German third-level Pediatric Surgery Departments. Biometric and periprocedural data were assessed. Main outcome measure was the feasibility of VAC therapy for giant omphalocele. Data are reported as median and interquartile range (Q1–Q3).

**Results:**

Gestational age was 37 (37–38) weeks, and birth weight was 2700 (2500–3000) g. VAC dressing was changed every 3 (3–4) days until abdominal fascia closure at the age of 9 (3–13) days. Time to first/full oral feeds was 3 (1–5)/20 (12–24) days with a hospital stay of 22 (17–30) days. Follow-up was 8 (5–22) months and complications were of minor extent (none: *n* = 2; Clavien–Dindo I: *n* = 3; Clavien–Dindo II: *n* = 1), comprising a delayed neo-umbilical cord rest separation (*n* = 2) and/or concomitant neo-umbilical site infection (*n* = 2) with no repeat surgery.

**Conclusion:**

In neonates with giant omphalocele, VAC constitutes a promising and technically feasible enhancement of the staged gravitational reduction method. This study shows evidence that VAC may accelerate restoration of the abdominal wall integrity in giant omphalocele, thus minimizing associated comorbidities inherent to a prolonged hospitalization.

## Introduction

Omphalocele (OC) represents the most common entity of congenital abdominal wall defects. In OC, eviscerated abdominal organs herniate through the umbilical cord which is covered by a three-layered membrane consisting of peritoneum, Wharton's jelly, and amnion. The extent of evisceration often correlates with the abdominal wall defect size and may comprise intestine and solid organs as liver, spleen, bladder, or gonads. The current prevalence of OC is 1.24 per 10.000 live births (1). The primary intention in OC treatment is a timely return of abdominal organs back into the peritoneal cavity and closure of fascial and skin defect without compromising the visceral and systemic perfusion. In contrast to the treatment of giant OC, which are generally defined by a defect size larger than 5 cm and/or a herniation of more than 50% of liver, minor OC may often be managed by primary closure (2, 3). Giant OC often requires gradual reduction of the external peritoneal viscera in order to minimize the risk for cardiopulmonary complications prior to either surgical tension-free abdominal fascial closure or a nonoperative closure allowing for epithelization of the OC sac with delayed fascial closure. Recently, vacuum (VAC) therapy has been introduced for the treatment of complicated cases in OC (4–11). However, VAC therapy has mostly been implemented after failure of another primary OC repair method with only one case series by Aldridge et al. (12) in which VAC therapy was utilized by primary intention within first two days of life as an adjunct to the staged gravitational OC reduction until complete abdominal reintegration of giant OC with time to fascial closure lasting several months. In the present study, we aimed for describing our experiences with the staged OC reduction approach in adjunct with primary vacuum application started within first hours of life.

## Patients and methods

### Patients

This study comprises a retrospective two-institutional chart review of six neonates born between September 2018 and May 2022 with giant omphalocele [International Classification of Diseases, 10th Revision (ICD-10-GM); code Q79.2] who received a staged gravitational reduction of extracoelomic OC contents in combination with VAC therapy at the Departments of Pediatric Surgery in Munich (Bavaria, Germany; *n* = 2) and Witten (North Rhine-Westphalia, Germany; *n* = 4). Any minor OC amenable to primary reduction was considered a criterion for exclusion. Main outcome measure was the feasibility of VAC therapy in OC patients. Secondary outcome measures were demographic or procedural parameters as time to first or full (defined as the entire nutritional calories obtained enterally) enteral feeds. All VAC procedures were performed under either the first or last authors' supervision; both of which were familiar with the technique. Follow-up investigation was performed either by outpatient consultation or by personal communication. For quantification of complications, the Clavien–Dindo Classification was applied, as described elsewhere (13). This classification consists of seven grades (I, II, IIIa, IIIb, IVa, IVb, and V) and objectifies the therapy necessary to correct a specific complication. In the study, only grade I and II complications were observed with grade I representing any postoperative course deviation from normal without further interventions (i.e., surgery with the exception of wound infections opened at the bedside) and grade II complications representing any complications requiring pharmacological treatment with drugs different to that allowed for grade I complications, including the use of antibiotics. This study was approved by the Ethics Committee of Ruhr-University Bochum (registry no. 22-7547-MPG, date of approval: 06/21/2022) and parental consent was obtained in each case.

### Methods

#### Technique

**Figure 1:**
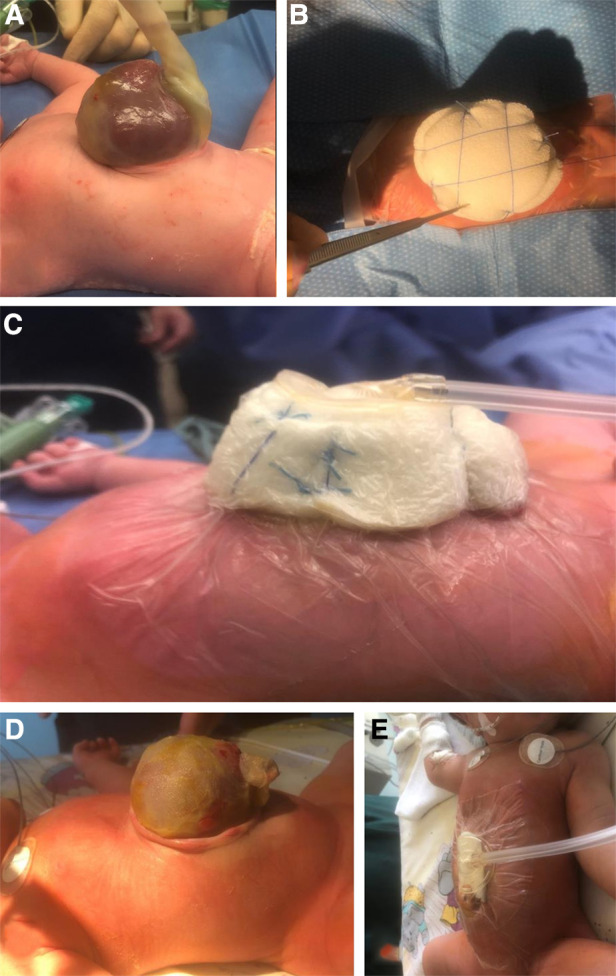
(**A**) Giant omphalocele with eviscerated bowel and liver. (**B**) Cutaneous sagittal and horizontal four-point-fixation of foam cylinder. (**C**) Lateral view on attached VAC device. (**D**) Omphalocele after removal of VAC dressing. (**E**) Lateral view at VAC device after amnial plication maneuver and foam application just above the skin level.

All patients included had prenatal diagnosis of OC and they were specially referred to the treating centers for delivery. Upon delivery, each patient was primarily taken care of by a consultant-level neonatologist and was immediately covered from neck downward with a sterile bowel bag (20″ ×  20″; Steri-Drape™; 3M Deutschland GmbH Health Care Business, Neuss, Germany) followed by elective intubation with subsequent mechanical ventilation. After induction of anesthesia, integrity of OC was either confirmed or restored, as in the case of sac rupture. The VAC technique consisted of the following steps:

(1) White Foam (Vivano®Med; Mondomed NV, HAMONT-Achel, Belgium) was attached to the OC surface (Figure 1A) in a cylindric shape after trimming the umbilical cord.
(2) Cutaneous sagittal and horizontal tight four-point-fixation (traction sutures) of the foam cylinder (Figure 1B).
(3) Cavilon™ no sting barrier film (3M Deutschland GmbH Health Care Business, Neuss, Germany) application to degrease the skin.
(4) RENASYS Transparent Film adhesive drape (Smith & Nephew Orthopaedics GmbH, Tuttlingen, Germany) was applied covering the entire lower torso including the foam cylinder. By this, kinking of OC with consecutive vascular structure occlusion was prevented.
(5) RENASYS Soft Port connector (Smith & Nephew Orthopaedics GmbH, Tuttlingen, Germany) was attached to the adhesive foil.
(6) RENASYS TOUCH Device (Smith & Nephew Orthopaedics GmbH, Tuttlingen, Germany) was activated and a permanent negative pressure of −80 mmHg was exerted (Figure 1C).

There was no discontinuation of VAC therapy other than during VAC dressing changes (Figure 1D). VAC dressing change intervals were chosen at the surgeons' discretion. Procedure of VAC changing included a further reduction of extracoelomic contents with the amnial plication maneuver and a complete coverage of OC surface with foam. In all patients, amnial plication of OC contents was performed under continuous cardiorespiratory monitoring. Definite closure of fascia was carried out when all OC contents were reduced within the abdominal cavity without cardiopulmonary compromise or signs of abdominal compartment syndrome. Closure included removal of the OC membrane at the fascial level with consecutive vertical midline closure of fascia using VICRYL™ 2–0 (Johnson & Johnson Medical GmbH, Ethicon Deutschland, Norderstedt, Germany) sutures and skin closure with either continuous subcuticular running sutures MONOCRYL™ 5–0 (Johnson & Johnson Medical GmbH, Ethicon Deutschland, Norderstedt, Germany) or LEUKOSTRIP^©^ S (4 × 38 mm; Smith & Nephew Orthopaedics GmbH, Tuttlingen, Germany). Neo-umbilicoplasty was performed at the lowest edge of the abdominal wall defect by preserving a 1–2 cm wide stripe of OC including the ligated umbilical arteries followed by a z-omphaloplasty, as adapted from Michel et al. (14). After abdominal wall closure, VAC therapy was continued at the skin level (Figure 1E) and then removed after 3–6 days.

#### Data analysis

Sampling and statistical analysis of data were performed using OriginPro 2021 (OriginLab, Northampton, MA, United States; RRID: SCR_014212). For descriptive statistics, the median and interquartile range was utilized. Categorical variables were presented as frequencies. The Kolmogorov–Smirnov test at a 0.05 significance level was used to confirm the normal distribution of numeric variables.

### Results

Six patients underwent VAC-assisted reduction of OC with a female preponderance (*n* = 5; 83%). Individual basic demographic and procedural characteristics are enlisted in Table 1 and Supplementary Figure S1. Gestational age was 37 (37–38) weeks. Birth weight, length, and head circumference were 2700 (2500–3000) g, 49 (47–49) cm, and 34 (32–35) cm, respectively. The amount of VAC dressing procedures was 3 (1–4) per patient with a dressing change interval of 3 (3–4) days and a VAC therapy duration was 9 (3–13) days until tension-free closure of the abdominal fascia and skin. First oral feeding was initiated on day 3 (1–5) of life and full oral feeding was achieved at the age of 20 (12–24) days. Length of stay (LOS) was 22 (17–30) days. All patients were primarily intubated with 5 (0–12) days on ventilation. Prophylactic antibiotics were administered in each case, as was the creation of a neo-umbilicus. In patient 3, OC membrane rupture was repaired by continuous sutures and a patent omphalomesenteric duct (OMD) was ligated at its base. Postsurgical complications are enlisted in Table 1. The follow-up period was 8 (5–22) months and main complications were associated with neo-umbilical formation, namely, a delay in umbilical cord rest drop off (*n* =2) and/ or umbilical site infection (*n* = 2) with no case needing repeat surgery. Specifically, complications at follow-up were nonexistent (*n* = 2) or graded Clavien–Dindo I (*n* = 3) or Clavien–Dindo II (*n* = 1), respectively. All patients demonstrated an age-appropriate neurodevelopmental status and their weight, height, and head circumferences increased along normal centiles.

**Table 1:** Characteristics of patients undergoing vacuum-assisted staged reduction of giant omphalocele.

Patient	1	2	3	4	5	6	All
Sex (M/F)	F	M	F	F	F	F	1/5
Gestational age (weeks)	38	37	37	37	38	37	37 (37–38)
Birth weight (g)	2,760	2,500	3,000	2,640	2,480	3,400	2,700 (2,500–3,000)
Birth length (cm)	46	47	49	48	49	52	49 (47–49)
Head circumference (cm)	35	31	34	33	35	32	34 (32–35)
OC content	B + L	B + L	B + L	L	B + L	B + L	—
VAC dressing changes (n)	4	2	4	1	3	1	3 (1–4)
Average VAC dressing change interval (days)	4	4	3	3	4	3	3 (3–4)
VAC duration until fascial closure (days)	17	7	10	3	13	3	9 (3–13)
Time to first oral feeds (days)	18	4	5	1	1	1	3 (1–5)
Time to full oral feeds (days)	28	22	18	11	24	12	20 (12–24)
Length of stay (days)	137	24	19	17	30	15	22 (17–30)
Initial tracheal intubation	Yes	Yes	Yes	Yes	Yes	Yes	—
Overall time on ventilation (days)	62	9	12	0.3	1	0.4	5 (0.4–12)
Average VAC dressing change duration per session (min)	29	39	49	18	58	28	34 (28–49)
Duration of fascial closure procedure (min)	111	91	129	59	86	56	89 (59–111)
Associated malformations	None	None	Cleft palate	None	VSD, PFO, PDA	Beckwith-Wiedemann syndrome	—
Complications during hospital stay	Respiratory insufficiency, central line septicemia	Liver bleeding and subileus after OC repair	Patent OMD and peripartal OC sac rupture	None	VAC-induced segmental liver infarction (increase in transaminases and CRP)	Initial feeding problems due to macroglossia	—
Main accompanied diagnosis	^a^	^b^	^c^	^d^	^d^	^e^	—
Prophylactic antibiotics	Yes	Yes	Yes	Yes	Yes	Yes	—
Neo-umbilicus formation	Yes	Yes	Yes	Yes	Yes	Yes	—
Follow-up period (months)	45	22	9	6	5	2	8 (5–22)
Disease specific complications at follow-up	None	Inclusion liver cyst at age of 2 years	Delayed umbilical cord rest drop off and omphalitis (iv antibiotics)	Delayed umbilical cord rest drop off	Suture string infection at umbilical site	None	—
Clavien–Dindo classification	None	I	II	I	I	None	—

M, male; D, female; OC, omphalocele; VAC, vacuum; B + L, bowel and liver; VSD, ventricular septal defect; PFO, patent foramen ovale; PDA, patent ductus arteriosus; OMD, omphalomesenteric duct; CRP, C-reactive protein.

Summarized and descriptive values are expressed as median (Q1–Q3).

^a^ Pulmonary hypoplasia (chronic respiratory insufficiency), recurrent pneumonia, scoliosis, and gastroesophageal reflux with gastrostomy placement.

^b^ Inguinal hernia, hydrocele testis, cutaneous haemangioma, and failure to thrive.

^c^ Temperature regulation disturbance, apnea bradycardia syndrome, and insufficient drinking.

^d^ Temperature regulation disturbance, insufficient drinking.

^e^ Insufficient drinking.

## Discussion

We present our preliminary experience with simultaneous VAC application as an improvement of the staged gravitational reduction technique in six patients with giant OC. No consensus as to the preferable surgical treatment of giant OC exists, which is mainly due to the heterogeneity of applied methods (15). Since its introduction in 1997 (16, 17), the vacuum technique has been subject to a large spectrum of applications in the adult population. However, it has not yet proven the same efficacy and safety in children (18). Nonetheless, the literature on the efficacy of vacuum therapy seems promising in terms of the treatment of complicated pediatric wounds as pressure ulcers, extremity wounds, surgical wound dehiscence, skin grafting, or complex abdominal defects (9–11, 19–21) and also congenital abdominal wall defects (5, 22). A review of the literature on VAC application associated with omphalocele revealed that VAC has mostly been implemented as secondary salvage therapy after failure of another primary OC repair method (4–11, 23) (Table 2). It is worth mentioning that reported cohort sizes were small ranging from one to three patients or were not reported at all (9, 11). Time to abdominal fascial closure ranged from 17 days to ∼29 months. Time to full enteral feeds was not documented in all cases, with only one exception (8). LOS ranged from 60 to 78 days (6–8, 12) or was also not reported (4, 5, 9–11, 23).

**Table 2:** Synopsis of literature reports on VAC application associated with omphalocele.

References	Sample size (*n*)	GA (weeks)	LOS (days)	VAC initiation	VAC duration (days)	VAC change frequency	VAC pressure (mmHg)	Time to first/ full feeds (days)	Time to fascial closure	Further information
Present study	6	37^a^	22^a^	1st day of life	9^a^	3 days	−80	3/20^a^	9 days	^b^
Aldridge et al. (12)	8	37^a^	70^a^	1st–2nd day of life (no later than 5th day)	68^a^	2×/week	−25 to −50	6/19^a^	8–9 months	^b^
Kilbride et al. (4)	3	34, 34, 37	n.s.	21 days, 5 months, 18 months	45, 22, 36	every 3–5 days	−50	n.s.	n.s.	^c^
McBride et al. (5)	3 (+1 GC)	37, 38, 37	n.s.	8 months, 15 months, n.s.	32, 13, n.s.	≤1×/week	−40 to −80	n.s.	∼10 months, ∼16 months, at ∼29 months no fascial closure	^c^
David et al. (6)	1	37	60	22	28	4 days	−40	n.s.	17 days	^c^
Tranh Tri et al. (7)	1 (+2 GC)	36	78	24 days	24	4×/24 days	−30	n.s.	Not closed at follow-up aged 14 months	^c^
Travassos et al. (8)	1	38	∼70	14 days	∼63	On day 3, 9, 12 (later n.s.)	−75	n.s./45	27 months	^c^
McCord et al. (9)	n.s.	n.s.	n.s.	n.s.	n.s.	n.s.	n.s.	n.s.	n.s.	n.s.
Stoffan et al. (10)	2	n.s.	n.s.	n.s.	n.s.	n.s.	n.s.	n.s.	n.s.	n.s.
Rentea et al. (11)	n.s.	n.s.	n.s.	n.s.	n.s.	n.s.	n.s.	n.s.	n.s.	^c^
Binet et al. (23)	3	Full terms	n.s.	11 days, 1 month, 13 days	8 day, n.s., 7 days	Case 1 and 2: 1×/7 days; case 3: n.s.	−10 to −50	n.s.	“Complete healing” at 3.5 months, 6 months, 5 months	^c^

GA, Gestational age; GC, gastroschisis; LOS, length of hospital stay; VAC, vacuum therapy; n.s., not stated.

^a^ Median.

^b^ VAC combined with staged procedure.

^c^ Failed alternative repair method with delayed start of VAC.

Presented procedure has only been reported once by another group (12) (Table 2) in an equivalent setting, characterized by eight patients of similar gestational age with start of VAC therapy within the first two days of life and comparable VAC change intervals, but with much longer duration until fascial closure (8–9 months vs. 9 days in our study) and LOS (70 days vs. 22 days in our study). Obtained durations until first and full oral feeding were comparable to the results obtained by Aldridge et al. (6 and 19 days vs. 3 and 20 days in our study). Noteworthy, in the present study, exerted vacuum levels were higher (−80 mmHg) than those used by Aldridge et al. (−25 to −50 mmHg). In this context, recommended negative pressure setting in congenital abdominal wall defects ranges from −50 to −75 mmHg (4, 24, 25). By vacuum levels exerted more positive than a certain threshold, an insufficient or decelerated reintegration of visceral contents could occur. This might be one explanation for the comparably fast reduction of eviscerated OC contents in our study compared to that by Aldridge et al. Of note, higher vacuum levels for OC reduction may also induce higher transient intra-abdominal pressure levels with increased risk for cardiorespiratory depression. However, we did not observe any signs of cardiorespiratory compromise within our continuously monitored cohort.

Comparing the different treatment strategies for giant omphaloceles (26–33) (Table 3) and the presented method, LOS was only shorter in one series by Morabito et al. (27), using the vertical cord traction followed by the compression reduction method. The time to full oral feeds of 20 days in our VAC method was slightly longer than in most of the comparative studies (27–30), or was not reported (26, 32, 33). With regard to time until fascial closure, only three groups (24) reported shorter periods than in the present study, ranging from 4 to 7 days utilizing vertical cord traction followed by compression reduction method (27), the delayed external compression reduction method (28), or the gravitational autoreposition suture method (31), respectively. In concordance with our data, Mehrabi et al. (29) also elicited a ventilation duration of 5 days by utilizing an intraperitoneal tissue expander and traction of abdominal muscles (camel-litter method) in six OC patients. In all other studies, durations of ventilation were longer, ranging from 7 to 11 days (27, 28, 30, 31), or were not even mentioned (26, 32, 33). Noteworthy, except one study by Abello et al. (33) utilizing a constructed silo with an adhesive hydrocolloid dressing in 40 neonates, neither of the reported studies had large sample sizes (ranging from 6 to 22 patients) and therefore associated malformations as pulmonary insufficiency may have large impact on average procedural parameters, as was the case in our first patient with pulmonary hypoplasia (PH) (Table 1). By excluding patient 1, median duration of ventilation decreased from 5 (0–12) days to 1 (0–9) day only. Over the course of the study, the individual ventilation duration decreased (supplementary Figure S1B), reflecting a possible learning curve. However, this has to be confirmed by larger studies.

**Table 3:** Literature data on biometric and procedural parameters for different treatment strategies regarding giant omphaloceles.

References	Sample size (*n*)	GA (weeks)	LOS (days)	Time to first/full feeds (days)	Time to fascial closure (days)	Time on ventilation (days)	Technique
Present study	6	37	22	3/20	9	5	VAC combined with staged procedure
Kogut and Fiore (26)	10	35.5	71	n.s.	14^b^	n.s.	serial taping
Morabito et al. (27)	19 (1 died)	36	11	n.s./6	4 (time to reduction)^c^	7	Vertical cord traction followed by compression reduction
Brown and Wright (28)	6	36.5^a^	30.5^a^	6.3^a^ after closure/18.8^a^	5.6^a^	7.1^a^	Delayed external compression reduction
Mehrabi et al. (29)	8 (6 OC+2 GC)	n.s. (range 4 days–13 years)	38^a^	n.s./14^a^	21^a^	5^a^	Camel-Litter method-intraperitoneal tissue expander and traction of abdominal muscles
Pacilli et al. (30)	12	38	42^d^	n.s./12^d^	26^d^	8^d^	Silo of Prolene mesh attached to the fascia; defect closure without opening the amniotic sac after sequential reduction.
Uecker et al. (31)	12	36^a^	63^a^	n.s./29^a^	7^a^	11^a^	GRAVITAS (gravitational autoreposition sutures)
Dörterler (32)	22	n.s.	35^a^	n.s.	n.s.	n.s.	Conservative and elastic bandages
Abello et al. (33)	40	38	26	n.s.	14.6	n.s.	Construction of a silo with an adhesive hydrocolloid dressing (Duoderm^Ⓡ^)

OC, omphalocele; GC, gastroschisis; GA, gestational age; LOS, length of hospital stay; VAC, vacuum therapy; n.s., not stated.

^a^ Quantitative data are presented as median or mean.

^b^ Primary defect closure without a patch in 6 cases and gortex patch application (covered by skin) in 4 cases (fascial closure in 3 cases at 70 days, 75 days, and 11 months of age.

^c^ Treated not surgically in 7 cases, treated surgically in 11 cases (uncomplicated primary closure in 7 cases and delayed/secondary closure in 4 cases).

^d^ Values presented for nine surviving neonates.

The presented VAC method combines some advantages, as being noninvasive in terms of OC sac preservation until fascial closure. Thus, the achieved intestinal nontouch procedure also diminishes risk for development of intra-abdominal adhesions. In addition, VAC application promotes a gentle pressure elevation by intra-abdominal dead space obliteration with a decreased risk for cardiorespiratory compromise. It is important to mention that the VAC method may reach its limits in those 36%–57% (34, 35) of cases with giant OC that present with associated pulmonary hypertension or PH. Since this condition may worsen with increasing abdominal pressure, a careful cardiorespiratory monitoring under reduction of OC contents is mandatory. Given that our method was well tolerated in patient 1 with PH, we do not consider this comorbidity an exclusion criterion at this early stage of experience.

In our series, the VAC method permitted an early enteral feeding. In general, VAC is supposed to minimize bacterial biioburden (24). Consecutive VAC changes might be performed under less invasive (awake caudal) modes of anesthesia without intubation at bedside, thus lowering the risk for associated side effects in this vulnerable cohort. Furthermore, the presented method can easily be learned and may be advantageous in case of complications, such as a patent OMD or an OC sac rupture as seen in patient 3. By reducing time until fascial closure, risks for associated morbidities inherent to a prolonged hospitalization in this delicate age group may be mitigated by presented VAC method. Finally, repair of OC with simultaneous neo-umbilicoplasty is of high relevance regarding the patient's satisfaction and self-identification (36–38). In general, an umbilicus located in the midline at two-thirds of the distance from the symphysis to xiphoid is considered cosmetically acceptable (39). In our series, the position of the neo-umbilicus was dictated by the umbilical arteries and thus at the anatomically correct position.

### Limitations

The main limitations of this preliminary report were the small cohort size and the limited follow-up period. As a consequence, randomized controlled trials comparing the presented VAC technique to other OC reduction techniques are needed.

## Conclusions

In neonates with giant OC, VAC constitutes a promising and technically feasible enhancement of the staged gravitational reduction method when primary closure seems questionable. Even in complicated cases with OC membrane defects or associated intestinal malformations, the presented technique seems to be safe and effective. Only in one previous series, VAC has been used in a similar manner for primary OC treatment, but with longer time until fascial closure. In summary, this study underlines the importance of VAC as an enhancement enabling an accelerated restoration of abdominal wall integrity in neonates with giant OC within their first 2 weeks of life, thus minimizing associated comorbidities inherent to a prolonged hospitalization. Therefore, we provide evidence that VAC is a promising technique to improve the treatment of OC and should be investigated further in future studies.

## Data Availability

The raw data supporting the conclusions of this article will be made available by the authors, without undue reservation.
