# Supplementary material for: Vacuum-assisted staged omphalocele reduction: A preliminary report
Source: Front Pediatr. 2022 Nov 24;10:1053568. doi: 10.3389/fped.2022.1053568 (PMC9730811; doi:10.3389/fped.2022.1053568)
Supplement: Supplementary file 1 [file Image1.pdf]

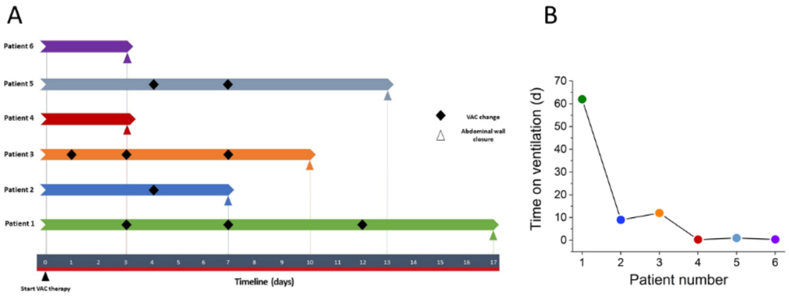

Figure S1. A) Individual timeline of patients undergoing vacuum-assisted staged reduction of giant omphalocele**. B)** Effect of decrease in individual ventilation duration over the course of the study.
